# Ranking of 10 legumes according to the prevalence of sensitization as a parameter to characterize allergenic proteins

**DOI:** 10.1016/j.toxrep.2021.03.027

**Published:** 2021-03-31

**Authors:** Mark Smits, Kitty Verhoeckx, André Knulst, Paco Welsing, Aard de Jong, Geert Houben, Thuy-My Le

**Affiliations:** a Division of Internal Medicine and Dermatology, University Medical Center Utrecht, Utrecht, the Netherlands; b Center for Translational Immunology, University Medical Center Utrecht, Utrecht, the Netherlands; c Netherlands Organisation for Applied Scientific Research (TNO), Utrecht, the Netherlands; d Julius Center for Health Sciences and Primary Care, University Medical Center Utrecht, Utrecht, the Netherlands; e Fresh Food & Chains, Wageningen Food & Biobased Research, Wageningen University, Wageningen, the Netherlands; f Netherlands Organisation for Applied Scientific Research (TNO), Zeist, the Netherlands

**Keywords:** EL, EUROLINE intensity units, ImpARAS, Improving Allergy Risk Assessment Strategy for New Food Proteins, SEM, standard error of the mean, IgE, immunoglobulin E, SPT, skin prick test, UMCU, University Medical Center Utrecht, Allergenicity prediction, Allergens, Legumes, Prevalence of sensitization, Ranking of allergens

## Abstract

Predicting the allergenicity of novel proteins is challenging due to the absence of validated predictive methods and a well-defined reference set of proteins. The prevalence of sensitization could be a parameter to select reference proteins to characterize allergenic proteins. This study investigated whether the prevalence of sensitization of legume extracts and proteins can indeed be used for this purpose. A random sample of suspected food-allergic patients (*n=*106) was therefore selected. 10 extracts (processed and non-processed) and 18 individual proteins (2S albumins, 7S and 11S globulins) from black lentil, blue and white lupine, chickpea, faba bean, green lentil, pea, peanut, soybean, and white bean were isolated and the prevalence of sensitization and the intensity of IgE binding were evaluated. The prevalence of sensitization ranged from 5.7 % (faba bean and green lentil) to 14.2 % (peanut). The prevalence of sensitization for individual legume proteins ranged from 0.0 % for albumin 1 (pea) to 15.1 %–17.9 % for Ara h 1, 2, 3, and 6 (peanut). The prevalence of sensitization correlated strongly with the intensity of IgE binding for individual proteins (*p* < 0.05, *ρ* = 0.894), for extracts no correlation was found. The discovered ranking can be used to select reference proteins for the development and validation of predictive *in vitro* or *in vivo* assays for the assessment of the sensitizing potential.

## Highlights

• 10 different legumes extracts could be ranked based on the variations in the prevalence of sensitization.
• Variations in the prevalence of sensitization allowed for ranking of 18 different individual legume proteins.
• Ranking can be used to select reference proteins to develop predictive assays for the assessment of the sensitizing potential of novel proteins.

## Graphical abstract

Patient serum from 106 suspected food-allergic patients was evaluated using line blots to investigate the prevalence of sensitization for legume extracts and individual legume components as a parameter to characterize allergenicity. A ranking was found based on the prevalence of sensitization and weakly and strongly sensitizing legumes were indicated.

**Figure:**
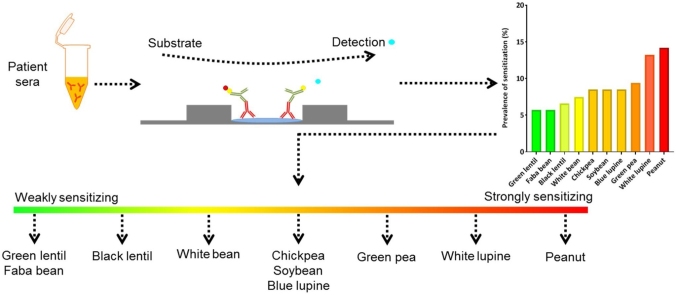

## 1 Introduction

Proteins constitute one of the four macronutrient groups in the human diet. A strong need of increasing the sustainability of the food protein supply is evident in view of climate change and population growth [[1], [2], [3], [4]]. Novel sustainable protein sources are therefore explored. However, introduction of novel protein sources onto the food market can pose a risk for allergic consumers due to their potential allergenicity [5,6]. In Europe, introduction of novel foods or foods processed with new techniques is subjected to laws and safety assessments as described in the General Food Law (Regulation EC no 178/2002). One of the important pillars of this legislation is that the introduction of novel food proteins does not add to the burden of food allergy [7]. Exposure to food proteins leads to tolerance induction but when this immune response fails, food allergy can develop [8]. The immune response in food-allergic patients can be divided in two phases: sensitization and elicitation. During the sensitization phase, specific immunoglobulin E (IgE) against a food protein is produced by B-cells [9]. Produced IgE then binds to the high affinity IgE receptor on mast cells and basophils, which are activated upon re-exposure with the food protein. This results in clinical complaints in the skin, gut, respiratory, and cardiovascular systems, with the severest form being anaphylaxis. A recent study by Lyons et al. estimated that the prevalence of probable food allergy in Europe ranged from 1.9 % to 5.6 % [10]. Sensitization is therefore an essential prerequisite for the development of food allergy and insight in the sensitizing potential of (novel) proteins could therefore be of great importance in assessing allergenicity.

All proteins can in theory induce sensitization and it is questionable whether non-sensitizing food proteins do exist [11]. On the other hand, it is likely that proteins vary considerably in their allergenic potency. Prediction of the allergenicity of novel food proteins is challenging and is currently based on the guidelines for genetically modified organisms established by the European Food Safety Authority [7]. According to these guidelines, characteristics such as sequence homology, binding of IgE from allergic individuals and stability of the protein are assessed in a weight-of-evidence approach. These methods have proven to be successful to predict potential cross-reactive allergy for novel foods such as chia seeds [7]. However, the same methods were not able to predict *de novo* sensitization and consequently a new allergy of novel food proteins of mealworm as described by Broekman et al. [12,13]. New or supplementary methods are therefore needed.

**Fig. 1:**
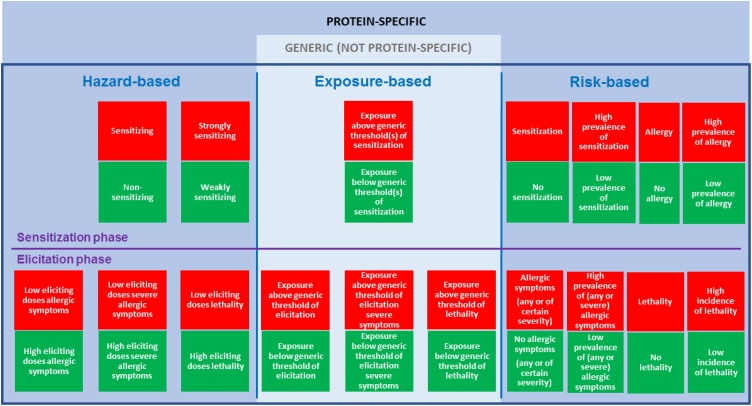
Overview figure of parameters and criteria for risk management decision-making. Overview of (theoretically) possible parameters/criteria (red and green boxes read horizontally across) for risk management decision-making with respect to IgE-mediated allergenicity of new or modified food proteins. Risk management decision-making could be based on a single parameter/criterion or on combinations of parameters/criteria. Green: an acceptable situation; red: a non-acceptable situation. Each (theoretically) possible option has specific implications for risk management and the methods and data needed for the assessment. Reprinted from: Defining the targets for the assessment of IgE-mediated allergenicity of new or modified food proteins, vol. number: 127, Houben G. et al., pages no. 61-9, copyright (2019) with permission from Elsevier [11].

It is important to come to a consensus on what these methods have to predict for (e.g. sensitization, allergic symptoms, severity of symptoms) before predictive methods for allergenicity assessment can be developed. Moreover, there is a need for a well-defined set of reference proteins (from weakly to strongly allergenic proteins) that can be used in the development of predictive methods. ImpARAS, a European COST Action (www.imparas.eu), discussed various parameters and criteria for allergenicity assessment as a possible step forward in risk management decision-making (Fig. 1) [11]. They differentiated distinct hazard-, risk- and exposure-based parameters and criteria for the sensitization and the symptom elicitation phase of food allergy. Each (theoretically) possible option has specific implications for risk management and the methods and data needed for the assessment. More information on the possible options and their implications can be found in the publication by Houben et al. [11]. The prevalence of sensitization was investigated in this study as a potential option. The prevalence of sensitization can be assessed, either qualitative (whether sensitization occurs or will occur) or quantitative (the prevalence of sensitization). Compared to other parameters for allergy (e.g. incidence of allergy or lethality), the prevalence of sensitization is a straightforward, solid and easy to measure parameter, though it must be noted that sensitization does not automatically lead to allergy. However, sensitization is a pre-requisite to cause an IgE-mediated food allergic reaction. Currently, the percentage of sensitized patients in an IgE-specific immunoassay determines if a protein is a minor (<50 %) or major (>50 %) allergen according to the FAO/WHO [14]. Ranking of proteins according to the prevalence of sensitization (low to high) could form the basis for the development of a ranking to partly characterize the allergenicity of novel food proteins. The ranking can be used to select reference proteins for the development and validation of predictive *in vitro* or *in vivo* assays and will be a step forward to support the development and application of allergenicity risk assessment approaches and methods.

To study if the prevalence of sensitization can be used to select reference proteins, a set of protein sources that include weakly and strongly allergenic proteins is needed. Some legumes like peanut, soybean, and lupine are particularly known for their allergenic potential. These three belong to the 14 foods responsible for an important part of food-allergic reactions and require mandatory labelling if used as an ingredient in food in the European regulatory region [15]. In contrast, allergic reactions to other legumes such as white beans and black gram are very rare [[16], [17], [18]]. The legume family is therefore an interesting group to investigate if proteins can be ranked according to the prevalence of sensitization. Apart from the difference in intrinsic allergenicity between legume proteins, processing is known to influence the IgE binding capacity of legumes as well and should be taken into account [19].

The objective of this study was to investigate differences in the prevalence of sensitization as well as the intensity of IgE binding of extracts and purified proteins of ten different processed and non-processed legume species (black lentil, blue lupine, chickpea, faba bean, green lentil, green pea, peanut, soybean, white bean, and white lupine) as a potential parameter to rank upon and select reference proteins for the evaluation of the sensitizing potential of novel food proteins.

## 2 Material and methods

### 2.1 Patient selection

Patients visiting the Allergology outpatient clinic at the University Medical Center Utrecht (UMCU) in the Netherlands between January and December 2018 with a suspected food allergy combined with a positive skin prick test (SPT) or prick to prick test (wheal size of ≥3 mm diameter), specific IgE in blood (≥0.1 kU/L on ImmunoCAP (ThermoFisher, Uppsala, Sweden) or ≥0.3 ISAC Standardized Units on ImmunoCAP ISAC 112 (ThermoFisher, Uppsala, Sweden)) were selected (*n* = 196). Included patients were at least 16 year old at time of screening. A random sample of *n* = 106 (from *n* = 196) was taken. Patients were evaluated using a line blot (EUROLINE, EUROIMMUN, Lübeck, Germany). In this study, residual material from diagnostic testing for research was used and was approved by the Biobank Research Ethics Committee of the University Medical Centre Utrecht (protocol number 18–428). The study was performed according to the principles of the Declaration of Helsinki.

### 2.2 Legume extracts and individual proteins

Black lentil (Lens culinaris - Canada), chickpea (Cicer arietinum - Turkey), faba bean (Vicia faba - Peru), green (French) lentil (Lens culinaris - Canada), green pea (Pisum sativum- The Netherlands), soybean (Glycine max - Canada), white bean (Phaseolus vulgaris - Canada), and white lupine (Lupinus albus - Germany) were obtained from www.peulvruchten.nl. Blue lupine (Lupinus angustifolius) was provided by the ScenoProt project, and peanut (Arachis hypogaea – United States) was obtained from www.de-eekhoorn.com. These commonly consumed legumes were selected based on their application as a sustainable protein source. Furthermore, selection of the aforementioned legumes ensured that legumes were included for which the prevalence of allergy ranged from high (for peanut and soybean) to low (white bean). Legumes were either non-processed or processed according to the supplier’s instructions before extraction. Some legumes were soaked and cooked (100 °C) in water and others were only cooked for a predetermined amount of time. An overview of the processing characteristics is given online (see Supplementary table 1). Peanuts were roasted in a hot air oven for 12 min at 175 °C. Legume seeds were grinded to a flour and sieved afterwards to remove lumps. Defatting was performed for 2.5 h at 120 °C by a Soxhlet extractor as reported by other publications, however, petroleum ether was used instead of diethyl ether [20,21]. Remaining contaminations of petroleum ether were removed using a vacuum stove for 48 h. Subsequently, the dried flour was sieved through a 0.5 mm sieve and extracted with 6 M Urea + 10 mM DTT (pH 8.0) for 1 h and centrifuged for 40 min. The supernatant was collected and stored at −80 °C until further use (legume extract).

Individual proteins were purified from non-processed legumes. The proteins were extracted using the Osbourne extraction in a procedure adapted from Freitas et al. [22]. In brief, legumes were grinded to a flour and sieved afterwards to remove lumps. After defatting by a Soxhlet extractor using petroleum ether for 24 h, albumins were extracted using an aqueous buffer (10 mM CaCl_2_, 10 mM MgCl_2_, pH 8.0) and centrifuged for 40 min at 7 °C. The supernatant containing the albumin fraction was collected. The pellet containing the globulins was suspended in 10 mM CaCl_2_ + 10 mM MgCl_2_ (pH 8.0) and centrifuged again for 40 min at 7 °C. The supernatant was discarded and the pellet containing the globulins was extracted using a high salt buffer (100 mM TRIS/HCl, 1 M NaCl, 10 mM EDTA and 10 mM EGTA, pH 8.2). The suspension was again centrifuged for 40 min at 7 °C. The supernatant containing the globulin fraction was collected. The globulin fraction was divided in a 2S albumin fraction and a 7S and 11S globulin fraction by using 100 kDa ultra-filtration. The individual proteins were further purified using anion-exchange chromatography under LPS free conditions after ammonium sulphate fractionation. Ammonium sulphate was added to the globulin fraction in a final concentration of 60 % or 70 %, depending on the protein. After centrifugation (20 min at 9000 rpm), the supernatants (containing the 7S globulins) were separated from the ammonium sulphate pellets (containing the 11S globulins). 3 mL of the supernatant was desalted using P10 columns equilibrated and eluted with 20 mM TRIS/HCl (pH 8.2) and 100 mM NaCl. Pellets were solubilized in 20 mM TRIS/HCl (pH 8.2) and 100 mM NaCl. The fractions were stored at −20 °C until further purification.

The proteins where further purified on a Source 15Q™column (GE Healthcare), which made LPS free by rinsing with1M NaOH, followed by LPS free Milli-Q. The column was equilibrated with three column volumes of buffer B (20 mM TRIS/HCl + 1.0 M NaCl pH 8.2) (max flow 60 mL/min), followed by three column volumes buffer A (20 mM TRIS/HCl pH 8.2). The samples (2.5 g protein), diluted with 50 mM NaCl in 20 mM TRIS/HCl (pH 8.2) to a concentration of 40 mg protein in 50 mL were loaded on the column and the column was washed with 2 column volumes buffer A. The proteins were eluted from the column using a linear gradient from 0 % to 60 % buffer B for 40 min (40 mL/min), followed by a linear gradient from 60 to 100 % B for 4 min. Fractions of 250 mL were collected.

The protein purity of all proteins was >95 %, except for Gly m 5 (>80 %), α-conglutin (>85 %), Pis s 1 (>90 %) and Legumin A (>90 %). Protein purity was measured using liquid chromatography–mass spectrometry, liquid chromatography with ultraviolet detection and sodium dodecyl sulphate–polyacrylamide gel electrophoresis. The identity of all proteins was confirmed using the same techniques. The following individual proteins were purified: α-conglutin, δ-conglutin, and Lup an 1 (blue lupine), Pis s 1, legumin A, albumin 1, albumin 2, and lipoxygenase (green pea), Ara h 1, Ara h 2, Ara h 3, Ara h 6, and Ara h 7.0201 (peanut), Gly m 5, Gly m 6, and lipoxygenase (soybean), and phaseolin and legumin (white bean).

### 2.3 Line blots

In total 38 extracts and proteins were placed on the EUROLINE line blot (EUROIMMUN, Lübeck, Germany). The line blot analysis was performed according to the manufacturer’s instructions. In brief, the line blot was incubated overnight with diluted (1:11) patient serum at room temperature on a rocking shaker in working strength universal buffer. Bound IgE antibodies were visualized using alkaline phosphatase-labelled anti-human IgE antibody and the substrate nitro-blue tetrazolium/5-bromo-4-chloro-3’s-indolyphosphate. The EUROLINE intensity units (EL) of the visualized bands were evaluated using the EUROLineScan software. The patient sera were negative for cross-reactive carbohydrate determinants. Patient sera was deemed positive if an intensity of 3 (class 1) or higher was found.

### 2.4 Data analysis and statistics

To calculate the sample size we used the Sample Proportion Simulation tool available at https://www.emathinstruction.com/sampleproportionsimulator/ to simulate the study. Based on our experience regarding legume sensitization in our outpatient clinic, a conservative expected population proportion of the lowest sensitizing legume (white bean) of 2.0 % was chosen. In combination with a power of 90 % and 1000 simulations, the calculated sample size was 106. A random sample of *n* = 106 (from *n* = 196) was therefore taken. Descriptive analyses were performed to report the sample proportion (%) of sensitization and intensity of IgE binding. For the intensity of IgE binding, the mean values with the standard error of the mean (SEM) were calculated. The Spearman’s rank correlation coefficient was used to investigate the correlation between the prevalence of sensitization and the intensity of IgE binding using SPSS Statistics 25 was used (IBM Corporation, Armonk, NY, USA). A *p*-value of <0.05 was considered statistically significant. Graphs were drawn using GraphPad Prism 8 (GraphPad Software, La Jolla, CA, USA).

## 3 Results

### 3.1 Prevalence of sensitization and intensity of IgE binding as potential parameters for the sensitizing potential of extracts

**Fig. 2:**
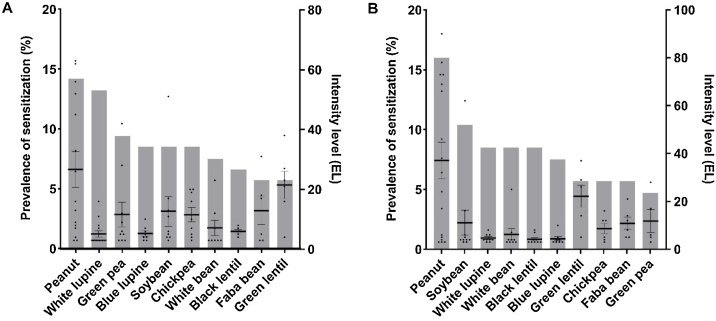
Sensitization and intensity of IgE binding of 10 legume extracts. The prevalence of sensitization and the intensity of IgE binding for 10 non-processed (A) and processed (B) legume extracts in a suspected food-allergic patient group (n = 106). The prevalence of sensitization (grey bars) is presented as a sample proportion and the intensity of IgE binding (black dots) are presented as the mean ± SEM.

The legumes sensitization profile of a random sample of 106 patients (mean age 35.1, range 16–75, 21.7 % male) visiting the Allergology outpatient clinic at the UMCU in 2018 with a suspected food allergy was evaluated. Fig. 2 shows the percentage of patients sensitized (bars) for the non-processed (Fig. 2A) and processed legume extracts (Fig. 2B) and individual intensity of IgE binding per patient (dots). The prevalence of sensitization for the non-processed legumes displayed sizable differences, which would indicate that ranking is possible. The highest prevalence of sensitization was seen for peanut (14.2 %), white lupine (13.2 %), and green pea (9.4 %), followed by blue lupine (8.5 %), soybean (8.5 %), chickpea (8.5 %), and white bean (7.5 %), and the prevalence was lowest for black lentil (6.6 %), faba bean (5.7 %), and green lentil (5.7 %). The intensity of IgE binding could be a potential additional parameter to rank upon, though it did not correlate with the primary parameter, i.e. the prevalence of sensitization. The Spearman’s rank correlation coefficient between the prevalence of sensitization and intensity of IgE binding was low (*ρ*=-0.183) and not significant (*p* > 0.05).

Legumes are generally processed before consumption, which may influence the allergenicity [19]. Sensitization for extracts, which were processed according to the supplier’s instructions, was therefore also evaluated to investigate the influence of processing on the ranking. Interestingly, an increase was seen in the prevalence of sensitization for peanut (16.0 %) and soybean (10.4 %), which are generally seen as strongly allergenic legumes. In contrast, the prevalence of sensitization for white lupine (8.5 %) and green pea (4.7 %) was decreased after processing. This indicates that processing influences the IgE binding of extracts and the resulting ranking. The Spearman’s rank correlation coefficient between the prevalence of sensitization and intensity of IgE binding for processed extracts was also low (*ρ*=-0.068) and not significant (*p* > 0.05).

### 3.2 Prevalence of sensitization and intensity of IgE binding as potential parameters for the sensitizing potential of individual proteins

**Fig. 3:**
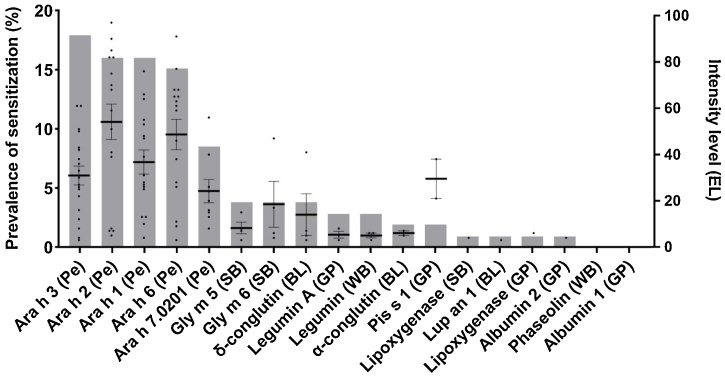
Sensitization and intensity of IgE binding of 18 individual legume proteins. The prevalence of sensitization and the intensity of IgE binding measured for 18 individual legume proteins in a suspected food-allergic patient group (n = 106). The prevalence of sensitization (grey bars) is presented as a sample proportion and the intensity of IgE binding (black dots) are presented as the mean ± SEM. BL, blue lupine; GP, green pea; Pe, peanut; SB, soybean; WB, white bean.

Fig. 3 shows the prevalence of sensitization for individual legume proteins. The highest prevalence of sensitization was seen for Ara h 3 (17.9 %), Ara h 1 (16.0 %), Ara h 2 (16.0 %), Ara h 6 (15.1 %), and Ara h 7.0201 (8.5 %) from peanut. Sensitization to the major allergens from soybean (Gly m 5 (3.8 %) and Gly m 6 (3.8 %)) was also frequently detected. The prevalence of sensitization for the major allergens from peanut and soybean was higher than from other established allergens such as Pis s 1 (1.9 %) from green pea and Lup an 1 (0.9 %) from blue lupine. Reactivity to phaseolin from white bean and albumin 1 from green pea was not detected. Mean intensity of IgE binding for 2S albumin peanut allergens Ara h 2 (54.1 AU) and Ara h 6 (48.6 AU) were higher compared to 7 S–11 S globulin allergens Ara h 1 (36.8 AU) and Ara h 3 (31.0 AU). Mean intensity of IgE binding for the major pea allergen Pis s 1 (29.5 AU) and the major allergens from soybean (Gly m 6 (18.5 AU) and Gly m 5 (8.3 AU)) were also high. In contrast to the legume extracts, the intensity of IgE binding of individual legume proteins correlated strongly and significantly with the prevalence of sensitization (*ρ* = 0.894, *p* < 0.05).

## 4 Discussion

This study showed that extracts and individual proteins from 10 different legumes could be ranked according to their prevalence of sensitization. This ranking could be used as a practical and straightforward parameter for various risk management decision-making processes. A ranking based on the prevalence of sensitization can also be used to select reference proteins for the assessment of the sensitizing potential of novel food proteins at which predictive method development can be targeted [7,11,23]. The results of the novel food protein in a biological assay can be compared to the reference set of weakly and strongly allergenic proteins to characterize the sensitizing potential of the novel food protein.

### 4.1 The prevalence of legume extract sensitization allows for ranking

Differences in the prevalence of sensitization between the total extracts of legumes were found in our study. The differences allowed for ranking of legumes from a high prevalence of sensitization (14.2 %) to legume extracts with a low prevalence of sensitization (5.7 %). A different ranking was seen when the legumes were heat processed. Processing (e.g. thermal treatment, hydrolysis, and fermentation) can affect the immunoreactivity of proteins and its effect on multiple foods has been reviewed [24]. For example, roasting increased the IgE binding of peanut allergens Ara h 1 and 2 compared to Ara h 1 and 2 that were isolated from raw peanuts [25]. Boiling did not influence the IgE binding capacity of lupine seeds [26], but did reduced the IgE binding capacity of peanut allergens [27]. Processing should therefore be taken into account. Differences in the prevalence of sensitization between legume extracts were also found by others [[28], [29], [30], [31], [32]]. A study by Kasera et al. investigated the prevalence of sensitization in India by means of SPT (*n* = 355) in patients with a history of legume allergy and discovered that kidney bean sensitization (22.0 %) was the most common legume to which sensitization was measured followed by chickpea (18.0 %), peanut (15.0 %), pigeon pea (11.5 %), black gram and green gram (11.0 %), soybean (9.5 %), green pea (6.7 %), lentil and cowpea (2.0 %). Geraldes et al. conducted a study in Portugal which evaluated the prevalence of sensitization of six legumes based on IgE titers (*n* = 13) in patients with a history of legume allergy and found the highest prevalence for peanut (71 %), followed by lupine (60 %), soybean (50 %), white bean (36 %), pea (33 %), and the lowest for chickpea (20 %).

The results from the studies of Kasera et al. and Geraldes et al. are in line with our results, showing that ranking of legumes according to the prevalence of sensitization was possible. However, both studies also showed differences in the prevalence of sensitization to the various legumes compared to our study. The relatively high prevalence of chickpea, pigeon pea, black and green gram sensitization compared to peanut sensitization in the study of Kasera et al. might be associated with the fact that these are staple foods in India, which increases the exposure to these foods and possibly the chance of developing sensitization [33]. Sensitization for green pea was ranked higher than soybean in our study compared to the study of Geraldes et al. Geraldes et al. evaluated sensitization in legume-allergic patients, which possibly explains the differences seen between the studies. Moreover, the study population of Geraldes et al. was small (13 patients) and sensitization was not determined for all legumes in all patients and subsequently the actual prevalence of sensitization to the various legumes could not be determined. In summary, ranking of legumes based on the prevalence of sensitization to extracts is possible, but it should be taken into account that geographical location, study population and processing could influence the prevalence of sensitization and the resulting ranking.

### 4.2 The prevalence of sensitization for individual proteins also allows for ranking

To the authors’ knowledge, this is the first study that investigated ranking of sensitization to multiple individual proteins from different legume species. Previous studies have focused on sensitization of individual proteins within one legume species (peanut or soybean), and little is known about the prevalence of sensitization to individual proteins of other legumes. Our study shows that the prevalence of sensitization for peanut proteins was more frequently seen compared to proteins from other legume sources. Ara h 3 sensitization was the most prevalent, followed by Ara h 2, Ara h 1, Ara h 6 and Ara h 7.0201. The peanut proteins were followed by Gly m 5 and Gly m 6 (soybean) and δ-conglutin (lupine). Sensitization to individual proteins from white bean (legumin and phaseolin) and green pea (legumin A, Pis s 1, lipoxygenase, albumin 1, and albumin 2) was less common. A previous study from Valcour et al. evaluated sensitization to peanut proteins (Ara h 1, 2, 3, 8, and 9), in 12,155 peanut-sensitized patients and found the highest prevalence for Ara h 2 (61.5 %), followed by Ara h 1 (43.2 %) and Ara h 3 (32.3 %) [34]. A similar ranking of Ara h 2 followed by Ara h 1 and Ara h 3 was found by others [[35], [36], [37], [38], [39], [40], [41], [42], [43]]. The difference in ranking based on the prevalence of sensitization in our study compared to other studies could be explained by the difference in study population. These studies evaluated peanut component sensitization in a peanut-allergic patient population. Ara h 2 and Ara h 6 were shown to be important diagnostic markers with a high prevalence of sensitization in peanut allergic patients [[44], [45], [46], [47]]. Subsequently, evaluation of these markers in a distinctly different population (in our study of suspected food-allergic patients) could lead to a different ranking. Co-sensitization between 11S globulins could have occurred in our suspected food-allergic population, in which patients are allergic to a multitude of foods compared to a peanut-allergic population. This could have led to the high prevalence of sensitization for Ara h 3. The prevalence of sensitization for 11S globulins (legumin-like) of peanut (Ara h 3), soybean (Gly m 6), white bean (legumin), and green pea (legumin A) was indeed higher than that of components of other protein families. Co-sensitization can occur between structurally homologous 11S globulins, which has been described for Ara h 3 and Gly m 6 [48,49]. Indeed, 75 % of Gly m 6 sensitized patients were also sensitized to Ara h 3 in our study (data not shown). This is in line with a study of Blankestijn et al. who found that 77 % of Gly m 6 sensitized patients were also sensitized for Ara h 3 [50]. A reference set of weakly and strongly allergenic proteins can be selected based on the ranking of individual proteins. This set of proteins can be used in the development and validation of predictive biological assays as mentioned by Mazzuchelli et al., who identified the absence of a reference set of proteins as a gap in the current allergenicity risk assessment [6]. The results obtained for novel food proteins in the biological assays can be compared with those from the allergenic reference set to be able to make statements about the possible sensitizing potential of the novel protein. In summary, the prevalence of sensitization for individual proteins can be used to rank and select a reference set of proteins that can be used to characterize the sensitizing potential of novel food proteins in predictive biological assays. However, study population and cross-reactivity can influence the ranking of individual proteins.

### 4.3 The intensity of IgE binding correlates with the prevalence of sensitization of individual legume proteins but not with extracts

IgE binding and its intensity can be influenced by several factors, such as the relative abundance of the allergens in the extract or binding of the allergens to other compounds in the extract [51]. This might explain why no correlation between prevalence of sensitization and the intensity of IgE binding was found for the extracts but in case of the individual proteins, correlation was strong. However, it must be noted that the correlations calculated for allergens for which only one or a few sensitized patients were found are not reliable due the limited number of data points. We therefore suggest using the prevalence of sensitization for ranking. This parameter is a more robust and straightforward parameter in comparison to the intensity of IgE binding.

### 4.4 Relationship between the ranking based on sensitization and clinical food allergy

We are aware that sensitization does not automatically lead to a food allergy, but sensitization is an essential pre-requisite for clinical food-allergic reactions and could therefore be an important parameter in allergenicity risk assessment. Previous studies have shown that a higher intensity of IgE binding resulted in a higher chance of an actual food allergy [[52], [53], [54]]. For instance Klemans et al. reported a 95–100 % positive predictive value of >5.0 kU/L for Ara h 2 IgE levels to diagnose peanut allergy. For peanut extract, IgE levels of ≥15 kU/L had a 95 % positive predictive value [47,55,56]. Our study showed that intensity of IgE binding for individual proteins correlates strongly with the prevalence of sensitization for individual proteins, making it likely that prevalence of sensitization also will correlate with the occurrence of food allergy. Food allergic reactions to peanut and soybean are commonly seen and investigated, whereas allergic reactions to other legumes (e.g. beans) are hardly reported [[16], [17], [18],^57^]. This implies that the prevalence of allergy for peanut and soybean is higher compared to other legumes, which was also reflected in our ranking of legume proteins. This further supports that the prevalence of sensitization for individual proteins and extracts is a reliable parameter to rank allergenic potency upon.

### 4.5 Future perspectives

For the first time, a large set of extracts and individual proteins from different legume species were studied simultaneously on sensitization profiles. The study showed that it is possible to rank proteins or protein sources based on the prevalence of sensitization as theorized by the ImpARAS COST Action [23,58]. However, the development of a ranking is complicated by differences in the prevalence of sensitization between populations, age, and countries. For example, the prevalence of sensitization for Ara h 9 (non-specific lipid transfer protein) was found to be higher in Spain (60 %) compared to a Swedish (14.3 %) or an American (7.7 %) peanut-allergic population [59]. Additionally, cross-reactivity between legumes and processing can influence the ranking. Therefore, the established ranking also needs to be validated in other countries and populations, and the effects of processing and cross-reactivity should be taken into account. Additionally, the consumption (or lack thereof) of the legumes may also have influenced the ranking. However, the amount of legume consumption only plays a minor role in the prevalence of sensitization as was previously reported by Smits et al. [60]. The ranking needs to be extended to include other plant sources, as well as animal sources. ImpARAS investigated various parameters and criteria for allergenicity assessment [11], though it is up to the risk managers and regulators to decide what allergenicity tests should predict for and what needs to be prevented (e.g. sensitization or elicitation, mild or severe allergic reactions) when novel protein sources are introduced.

In this study, we evaluated the prevalence of sensitization as part of the allergenicity assessment and found a ranking based on the prevalence of sensitization that corresponds with the prevalence of legume allergy in the clinic. We hope that these findings will help risk managers and regulators to decide on a viable parameter to aid the development of methods for allergenicity assessment and defining a set of reference proteins from weakly to strongly sensitizing proteins.

## 5 Conclusions

The prevalence of sensitization is an interesting parameter to rank upon. Proteins with a high prevalence of IgE binding could be classified as a strong sensitizer and proteins with a low prevalence could be classified as a weak sensitizer. The ranked legume proteins from this study can be selected as reference proteins for the development and validation of predictive *in vitro* or *in vivo* assays for the assessment of the sensitizing potential of novel legume proteins. However, evaluation of other characteristics (e.g. study population age, processing, geographical location, other protein sources) is needed to confirm the value of ranking as part of the allergenicity assessment and risk managers and regulators need to decide if the prevalence of sensitization is an acceptable parameter to rank upon.

## CRediT authorship contribution statement

**Mark Smits:** Data curation, Formal analysis, Investigation, Visualization, Writing - original draft. **Kitty Verhoeckx:** Conceptualization, Funding acquisition, Project administration, Supervision, Writing - review & editing. **André Knulst:** Conceptualization, Supervision, Writing - review & editing. **Paco Welsing:** Methodology. **Aard de Jong:** Resources, Writing - review & editing. **Geert Houben:** Conceptualization, Funding acquisition, Supervision, Writing - review & editing. **Thuy-My Le:** Conceptualization, Supervision, Writing - review & editing.

## Declaration of Competing Interest

The authors declare no conflict of interest.
