# Supplementary material for: Ranking of 10 legumes according to the prevalence of sensitization as a parameter to characterize allergenic proteins
Source: Toxicol Rep. 2021 Mar 31;8:767–73. doi: 10.1016/j.toxrep.2021.03.027 (PMC8027524; doi:10.1016/j.toxrep.2021.03.027)
Supplement: Supplementary file 1 [file mmc1.docx]

**Supplementary Table 1**. **Processing characteristics of the legumes.**

| **Legume** | **Soaking time** | **Cook time** |
| --- | --- | --- |
| Black lentil (*L. culinaris*) | No | 20 minutes |
| Blue lupine (*L. angustifolius*) | Overnight | 60 minutes |
| Chickpea (*C. arietinum*) | 8 hours | 45 minutes |
| Faba bean (*V. faba*) | 8 hours | 45 minutes |
| Green lentil (*L. culinaris*) | No | 20 minutes |
| Green pea (*P. sativum*) | 8 hours | 50 minutes |
| Soybean (*G. max*) | 6 hours | 40 minutes |
| White bean (*P. vulgaris*) | 8 hours | 45 minutes |
| White lupine (*L. albus*) | 24 hours | 100 minutes |

Legumes were processed according to the supplier’s instructions before extracts were made. Legumes were soaked for up to 24 hours prior to cooking. Cooking time ranged from 20 minutes to 100 minutes.
